# Structural Changes of Oak Wood Main Components Caused by Thermal Modification

**DOI:** 10.3390/polym12020485

**Published:** 2020-02-21

**Authors:** Ivan Kubovský, Danica Kačíková, František Kačík

**Affiliations:** Faculty of Wood Sciences and Technology, Technical University in Zvolen, T.G. Masaryka 24, 960 01 Zvolen, Slovakia; kacikova@tuzvo.sk (D.K.); kacik@tuzvo.sk (F.K.)

**Keywords:** oak wood, thermal treatment, degradation, infrared spectroscopy, size-exclusion chromatography

## Abstract

Thermal modification of wood causes chemical changes that significantly affect the physical, mechanical and biological properties of wood; thus, it is essential to investigate these changes for better utilization of products. Fourier transform infrared spectroscopy and size exclusion chromatography were used for evaluation of chemical changes at thermal treatment of oak wood. Thermal modification was applied according to Thermowood process at the temperatures of 160, 180 and 210 °C, respectively. The results showed that hemicelluloses are less thermally stable than cellulose. Chains of polysaccharides split to shorter ones leading to a decrease of the degree of polymerization and an increase of polydispersity. At the highest temperature of the treatment (210 °C), also crosslinking reactions take place. At lower temperatures degradation reactions of lignin predominate, higher temperatures cause mainly condensation reactions and a molecular weight increase. Chemical changes in main components of thermally modified wood mainly affect its mechanical properties, which should be considered into account especially when designing various timber constructions.

## 1. Introduction

Wood is a sustainable and environmentally friendly natural material used for both structural and non-structural applications. It has excellent mechanical properties. However, one of the main disadvantages of wood is its hygroscopicity causing dimensional instability. Particularly the outdoor utilization of wood is highly limited by its strong hygroscopicity and low durability [1,2]. A thermal modification is also used in addition to the chemical modification to improve the dimensional stability of wood. Thermal modification (TM) is environmentally friendly and one of the most effective pretreatment methods to enhance the dimensional stability and decay resistance of wood without using any toxic chemicals. The heat applied ranges between 160 to 260 °C in a vacuum, nitrogen, air or oil environments [3,4]. Thermal modification causes changes in the structure of wood cell wall polymers, which is reflected in a change in its properties. Heat treatment conditions have a direct effect on the chemical decomposition of wood [5]. Wood is a complex biological material and the thermal decomposition of its main components (hemicellulose, cellulose and lignin) occurs through a series of chemical reactions. The modified wood composition results in a lower hygroscopicity with a major influence on both dimensional stability and durability [6]. It appears that the higher the treatment temperature, the better the durability, stability and biological properties of the product become. However, it should be noted that the overall mechanical properties of wood are weakened under such conditions [7,8]. The most sensitive of the structural components to thermal degradation are hemicelluloses. Acetic and formic acids arising during thermal treatment enhance hydrolysis of hemicelluloses and cellulose. The degree of polymerization of polysaccharides decreases, hydrolyzed sugars are further dehydrated, and a wide variety of volatile compounds is formed, such as formaldehyde, furfural, hydroxymethylfurfural and other aldehydes [9,10]. Cellulose crystallinity increases with thermal modification due to the degradation of its amorphous part, which is related not only to the temperature but also to the length of thermal treatment [11,12].

The chemical changes that occur in wood heat treatment process are affected by many external conditions (temperature, heating time, type and composition of the surrounding atmosphere). It is important to note that many competitive reactions are taking place simultaneously and are significantly influenced by the conditions of the thermal treatment. This is one of the reasons why several authors have come to different results in the investigation of heat-treated wood experiment.

**Figure 1:**
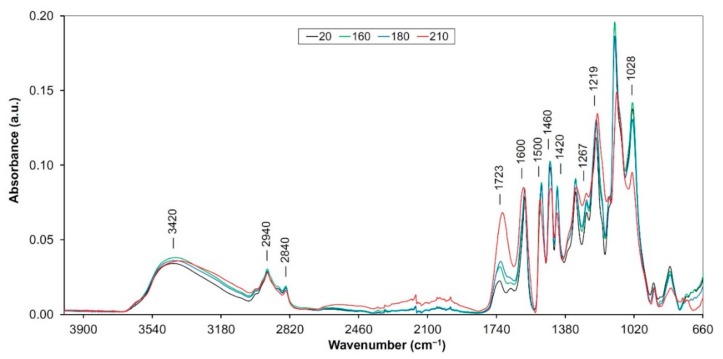
FTIR spectra of the thermally treated lignin from oak wood.

**Figure 2:**
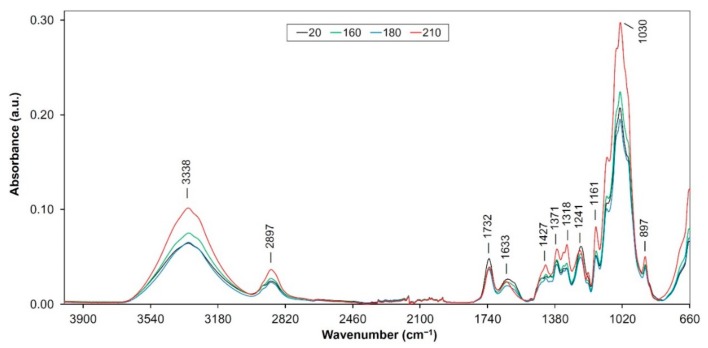
FTIR spectra of the thermally treated holocellulose from oak wood.

**Figure 3:**
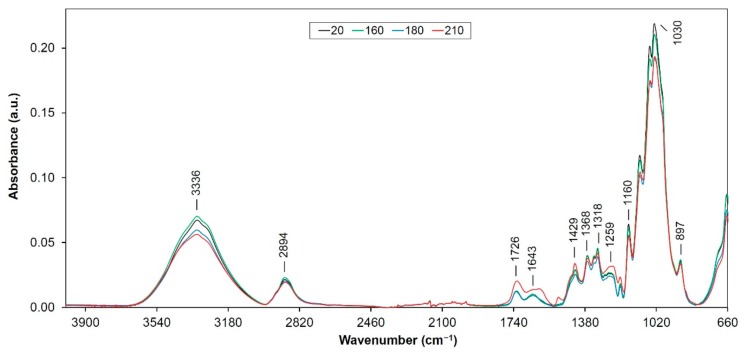
FTIR spectra of the thermally treated cellulose from oak wood.

As assumed the thermal treatment process of wood causes modifications and degradations in its main components. These processes occur through various reactions such as dehydration, hydrolysis, oxidation, decarboxylation and trans-glycosylation [13]. Several changes were observed in the FTIR spectra (Figure 1, Figure 2 and Figure 3), which were assigned to changes in the hemicellulose and lignin structures. Changes in the thermally treated oak wood, related to color and mechanical changes were described and analyzed in detail in several authors’ works [4,14,15]. In our study, we will focus on changes in main oak wood components isolated (extracted) from individual thermally treated samples.

The goal of this study is to examine the chemical changes of oak wood main components by Fourier-transform infrared spectroscopy (FTIR) and size exclusion chromatography (SEC), in thermally modified wood in order to clarify the processes of structural changes in wood polymers better.

## 2. Materials and Methods

### 2.1. Samples Preparation

The experimental material consisted of samples of European oak (*Quercus robur*, L.) in dimensions of 200 mm × 100 mm × 20 mm (longitudinal × tangential × radial). All samples were air-conditioned under specific conditions (RH 65% ± 3% and temperature 20 ± 2 °C) for more than 6 months to achieve an equilibrium moisture content (EMC) of 12%. After conditioning procedure were selected forty samples without visible defects and divided into four groups (each includes of ten samples). The first group served as a reference and was not heat treated. The remaining three groups were thermally modified at atmospheric pressure using three peak temperatures (160, 180, and 210 °C) according to [4]. The entire thermal treatment process involves three phases. The first phase concerns fast preliminary heating up to ca 100 °C followed by a slower temperature increase to 130 °C, the second phase represents slow heating period to 160–210 °C for 2–3 h and during third phase is wood cooled and its moisture content is stabilized on about 4–7%. Depending on the temperature of heat treatment these groups were further referred as “20” (untreated), “160”, “180” and “210”. The heat treated and untreated samples were then mechanically disintegrated and milled to a particle size of 200–300 μm using a POLYMIX PX-MFC 90D laboratory mill (Kinematica, Luzern, Switzerland) and dried (4 h at 103 ± 2 °C). They were then extracted in the Soxhlet apparatus (Sigma-Aldrich, Munich, Germany) using ethanol-toluene solution (1.0/0.427 v/v). Cellulose and holocellulose were obtained according to the procedures described in [4]. Dioxane lignin was isolated from each sample (10 g from 200 mL of dioxane-water mixture for 5 h at 80 °C) according to the procedures presented in [16]. Samples of cellulose, holocellulose and dioxane lignin were further analyzed by attenuated total reflectance (ATR)-FTIR and SEC techniques. The methods used to prepare and isolate the main wood components ensure their minimal contamination by substances from other wood components and can be considered clean and suitable for analysis.

### 2.2. ATR-FTIR Analysis

FTIR spectra of isolated main wood components were recorded on a Nicolet iS10 FT-IR spectrometer (Thermo Fisher Scientific, Waltham, MA, USA), equipped with Smart iTR using an attenuated total reflectance (ATR) sampling accessory attached to a diamond crystal. Initially, background measurement was performed to subtract it from the resulting spectrum. The spectra were acquired by accumulating 64 scans at a spectral resolution of 4 cm^−1^ in an absorbance mode from 4000 to 650 cm^−1^ and standardised using the baseline method. Obtained data were analyzed using the OMNIC 8.0 software. Measurements were performed four times per sample. 

### 2.3. Size-Exclusion Chromatography

The molecular weight distribution (MWD) of lignin was measured with Agilent 1200 HPLC chromatograph (Agilent Technologies, Santa Clara, CA, USA) by previously described SEC method [16]. The isolated dioxane lignin was dissolved in dimethyformamide (DMF) (c = 5 mg·mL^−1^) and filtered through a Puradisc 25 NYL filter (Whatman International, Maidstone, UK) with a pore size of 0.45 µm. The separation was performed at 35 °C with LiBr (0.005 M) in DMF at a flow rate of 1 mL·min^−1^ on a POLAR-M column (7.5 mm × 300 mm, Agilent Technologies, Santa Clara, CA, USA). A differential refractometer (RI) and diode array detector (DAD) at 280 nm were used as the detectors. Data were acquired with Chemstation software (Agilent) and analyzed with the Clarity GPC module. The system was calibrated with polystyrene standards from 500 to 98,900 g·mol^−1^. All SEC results represent the mean of two different samples and each sample was run in two replicates.

Samples of cellulose and holocellulose were derivatized using phenyl isocyanate to obtain tricarbanilates which were dissolved in tetrahydrofuran prior to the size exclusion chromatography (SEC) analysis. The derivatives were prepared according to procedure described previously [17]. SEC analyses were performed at 35 °C with tetrahydrofuran at a flow rate of 1 mL·min^−1^ on a two PLgel, 10 μm, 7.5 × 300 mm, MIXED B columns preceded by a PLgel, 10 μm, 7.5 × 50 mm, GUARD column (Agilent Technologies, Santa Clara, CA, USA). Two tricarbanilate derivatives were prepared for each sample and each derivative was analyzed twice.

## 3. Results and Discussion

### 3.1. Changes in the FTIR Spectra

As it is well known, the absorption bands characteristic of lignin, cellulose and hemicelluloses lie in the wavelength range from 1800 to 800 cm^−1^ (stretching and bending vibrations within the molecules, also called as the fingerprint region). In addition, an area between 3550 and 2900 cm^−1^ (OH and C−H stretching) is also important for the main wood components.

#### 3.1.1. FTIR Spectra of Lignin Isolated from Heat Treated Samples

The major lignin bands in lignin structure are approximately at 1596, 1510, 1464, 1423, 1367, 1326, 1269 and 1221 cm^−1^ [18]. Cheng et al. [19] investigated identifying changes in the main components of wood after thermal treatment, focusing on selected bands ranging from 1730 to 1110 cm^−1^. In addition, lignin can also be assigned a broad region including an interval 3300–3600 cm^−1^ (intramolecular hydrogen bond in phenolic groups, OH stretching of alcohols, phenols, acids and weakly bounded absorbed water) and bands about 2900 cm^−1^ (C−H stretching in methyl and methylene groups) [20,21,22]. The following changes indicate the thermal degradation of lignin that was isolated from the heat-treated oak samples.

In our case, at the 3420 cm^−1^ region only small changes with a slight decreasing of band intensities were observed (on samples “180” and “210”, Figure 1). Other researchers found a decrease in the amount of OH groups in the heat-treated wood too, probably due to condensation reactions [21,23]. The shift to lower wavelengths and mild widening of the band is also observable, which can be caused by oxidation and hydrolysis of acetyl groups from hemicelluloses [21] or crosslinking of free hydroxyl groups [24]. A similar trend was also seen in the 2940 cm^−1^ (asymmetric CH_2_ stretching) and 2840 cm^−1^ (symmetric CH_2_ stretching) band region (Figure 1). That trend might be due to structural and relative composition changes, namely, changes at the cellulose crystallinity level [25].

The absorption in FTIR spectra including an interval between 1750 and 1700 cm^−1^ (C=O stretching in unconjugated groups) reflects changes in various functional groups in lignin and hemicelluloses (carbonyls, ester groups, ketones, aldehydes, carboxylic acids) [21,26]. Our measurements showed an increase in absorbance on the 1723 cm^−1^ band (Figure 1). Its value copied the increase in temperature used in the heat treatment and band shifted to lower wavenumbers (from 1723 to 1708 cm^−1^). This shift may be due to conjugation of the carbonyl group to other double bonds (aromatics, alkenes) supported by an increase in carbonyl or carboxyl groups due to oxidation reactions as a result of the temperature increase in the thermal degradation process [21]. Li et al. 2002 [27] studied the heat degradation of lignin in hardwood and softwood and obtained an increase in the band at 1720 cm^−1^ with increasing temperature, which they concluded to be due to the production of C=O bonds in lignin. Increasing of intensity may be due to more pronounced cleavage of the *β*–alkyl–aryl ether bonds and the production of C=O bonds within the lignin [21,28]. Increasing the amount of acetyl groups and carboxylic acid groups from lignin and saccharides may also be the cause of absorbance growth [21,28]. The cause of these changes is the splitting of aliphatic side chains in lignin and cleavage of *β*–O–4 linkages, during thermal treatment [29,30].

The band near 1600 cm^−1^ (C=C stretching of the aromatic ring in lignin) is related to unsaturated linkages and aromatic rings present in lignin [31]. With increasing temperature initially, it slightly increases (but then shows only minor changes, Figure 1). The changes in this area are related to lignin condensation at the expense of conjugated carbonyl groups and to the carboxylation of polysaccharides [32].

The band at 1500 cm^−1^ (C=C aromatic skeletal vibrations stretching of the benzene ring in lignin) shows a weak decrease with increase of treatment temperature and shift to 1512 cm^−1^ (Figure 1). This band is associated with guaiacyl and syringyl units in wood lignin. A decrease in absorbance at higher temperature is due to the decrease of methoxyl groups, the loss of syringyl units or breaking of aliphatic side-chains [21]. Several authors reported the increase of absorbance around 1505 cm^−1^ in thermally treated beech, teak and oak wood [33]. Demethoxylation at thermal treatment is supported by the shifting of maximum absorption from 1505 cm^−1^ to 1512 cm^−1^ [34,35]. This trend was also observed by other authors [36,37].

At bands of 1460 cm^−1^ (asymmetric C−H deformations in lignin) and 1420 cm^−1^ (aromatic skeletal vibration in lignin with C−H deformation and carbohydrates) a slight decrease was noted (most sharply at 210 °C, Figure 1). These changes were caused by lignin degradation and the cleavage of methoxyl groups during heat treatment [16]. During the thermal treatment of lignin, there occurs elimination of water and methanol to give the conjugated ethylenic bonds [38]. The bands at 1267 cm^−1^ (C−O stretching of guaiacyl ring) [18] and at 1219 cm^−1^ (C−O stretching of syringyl ring) after an initial increase, they decrease slightly on all samples (Figure 1). However, the decrease in absorbent band intensity corresponding to the guaiacyl structure is milder than that of syringyl. The observed course confirms the assumption that the degradation of the syringyl units occurs at a lower temperature than guaiacyl [30]. According to [39,40], the bands are in the 1190 to 950 cm^−1^ area attributed to the C−O and C−H vibrations that are derived from aliphatic −CH_2_ or phenol −OH bonds. The slight decrease of absorbance at this region indicated gradual degradation of methyl and hydroxyl groups [2]. In our case the band at 1028 cm^−1^ (methoxyl groups in lignin) [41] decreased permanently (Figure 1). This trend is attributable to the partial demethoxylation of lignin and its gradual crosslinking [28].

#### 3.1.2. FTIR Spectra of Holocellulose Complex Isolated from Heat Treated Samples

The holocellulose (hemicelluloses + cellulose) degraded at the thermal treatment. Similarly, to the isolated dioxane lignin, there are striking bands around the carbohydrate complex around of 3300 cm^−1^, 2890 cm^−1^ and the band within the range of 1740 to 1725 cm^−1^. Bands at 3338 and 2897 cm^−1^ were increased with thermal modification process (Figure 2). This effect may be due to oxidation and hydrolysis of acetyl groups from hemicelluloses [21].

Band at 1732 cm^−1^ (Figure 2) is attributed to unconjugated carbonyl stretching in hemicelluloses (C–O stretching vibration in acetyl, carbonyl and carboxyl groups) [42]. Continual decreases in the signal intensities were observed together with an increase of treated temperature on all samples (Figure 2). This suggests that cleavage of acetyl side chains in hemicellulose occurred [43]. The decrease in absorbance around 1740 cm^−1^ could be due to reduced hemicellulose content in the heat-treated wood. Similar results were also obtained by [10,44] on heat-treated samples. Özgenç et al. 2017 [30] discovered that thermal treatment of wood induced the degradation of hemicellulose. Band at 1633 cm^−1^ (conjugated C−O in quinones coupled with C=O stretching in various groups) [32,43] shows a fluent though slight decrease under the thermal treatment (Figure 2). This trend may point to the cleavage of the α–alkyl–aryl ether bonds [16]. Absorbance at 1600 cm^−1^ and around of 1500 cm^−1^ have a minimal value and continually decreases, confirming the negligible content of benzene nuclei in isolated holocellulose (Figure 2). The bands around of 1427 cm^−1^ did not show any significant changes in the heat-treated samples (except the sample “210”, Figure 2). Absorption band at 1371 cm^−1^ (CH_2_ bending in cellulose and hemicelluloses) rose slightly at all samples (but most significantly at the sample treated at 210 °C, Figure 2). We found an increase of intensity around the 1318 cm^−1^ (Figure 2). According to [45,46], the higher intensity confirms an increase of condensed structures.

The band at 1241 cm^−1^ (C−O stretching vibration in xyloglucan) indicated a permanent decrease (Figure 2) in the height which again confirms the existence of more condensed structures [46]. Band at 1161 cm^−1^ (C−O−C vibrations in cellulose and hemicelluloses) [47] shows a slight increase, especially at 210 °C, (Figure 2). This behavior suggests dehydration reactions forming covalent intermolecular bonds, i.e., cross-links, by ether bonds and gradual decomposition of carbohydrates [25,48].

Bands at 1030 cm^−1^ (C−O−C stretching of primary alcohol in cellulose and hemicelluloses) [45,49] and 897 cm^−1^ (C_1_–H deformation of glucose ring in cellulose and hemicellulose) [31,50] increased continuously (Figure 2). It confirms gradual changes in the cellulose structure.

#### 3.1.3. FTIR Spectra of Cellulose Isolated from Heat Treated Samples

At the beginning it is necessary to consider the fact that in the method used, cellulose is not obtained in a pure state but only as a preparation with a small portion of lignin and hemicelluloses. FTIR spectra of cellulose are slightly perturbed by bands derived from lignin and hemicellulose. Band around 3336 cm^−1^ (intramolecular hydrogen bonds in cellulose) shows a gradual decrease in absorbance, where the highest decline shows at the samples modified at maximum temperature (Figure 3). This may be due to the lower content of hydroxyl groups in both holocellulose and lignin as a result of the process used to isolate cellulose from heat treated wood samples. At the band of 2894 cm^−1^ (C–H stretching in methylene groups), it was observed that the absorbance value decreases slowly (Figure 3). The cause of the decrease in absorbance may be cellulose degradation and an increase in its crystalline fraction [25].

The absorption bands of 1726 and 1643 cm^−1^ have a minimum height in the FTIR spectrum of isolated cellulose from thermally modified wood (Figure 3). These bands are associated with the hemicellulose complex, and their decline suggests decomposition of the carbohydrate components that have remained in the extraction of cellulose from the original wood samples.

The band around of 1429 cm^−1^ (C–H vibration in plane cellulose) [51] show small increase in absorbance with an increase of temperature (especially at “210” sample, Figure 3). It can be a confirmation of the increase in the amount of the crystalline cellulose. Absorption band at 1368 cm^−1^ (CH_2_ bending in cellulose and hemicelluloses), identically to the band at 1318 cm^−1^ decreased slightly, which confirms degradation of hemicelluloses (Figure 3). Conversely, the absorption of the band at 1259 cm^−1^ increased slightly, which may be due to residual lignin contained in the isolated cellulose. Intensity at 1160 cm^−1^ (C−O−C vibrations in cellulose) as well as 1030 cm^−1^ (associated with cellulose deformations) decreased gradually (Figure 3), probably due to beginning of cellulose degradation processes.

The band at 897 cm^−1^ which is specific to glucose ring stretching vibration decreased slightly with an increase of treatment temperature (Figure 3). It may be due to thermal degradation of *β*–(1,4) glycosidic bonds [37]. Decreasing the absorption at this band indicates a decrement of the amorphous form of cellulose [52].

### 3.2. Changes of Macromolecular Traits in Lignin and Polysaccharides

**Figure 4:**
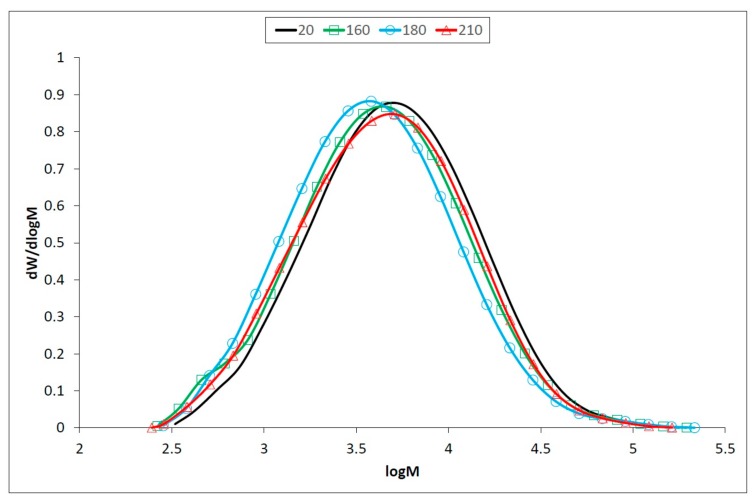
Molecular weight distribution of oak wood lignin before and after thermal treatment.

Lignin is one of the less thermally stable polymers, and its relative content increases after thermal treatment [53,54]. However, structural changes in the lignin during the modification, such as cleavage of the methoxyl groups and depolymerization of the lignin macromolecule to lower molecular weight compounds and subsequent re-polymerization. SEC analyses indicate (Table 1, Figure 4) a slight decrease of lignin molecular weight up to temperature of 180 °C, followed by its increase. The drop of molecular weight is due to cleavage of the different C−O bonds of C3 side chain and especially of *β*−O−4 ether linkage [5]. Simultaneously, reactive intermediate species like carbonium ions, which can be formed during cleavage of benzylic C−O bond, are involved in recondensation reactions [55]. 

The predominance of degradation and condensation reactions depends mostly on the temperature and the duration of the thermal treatment. At lower temperatures and/or at shorter modification, degradation reactions predominate. Higher temperatures and/or prolonged treatment cause mainly condensation reactions and molecular weight increases [56,57]. Our findings confirmed that depolymerization, side chain cleavage and recondensation occur during the exposure to heat [13,58].

**Figure 5:**
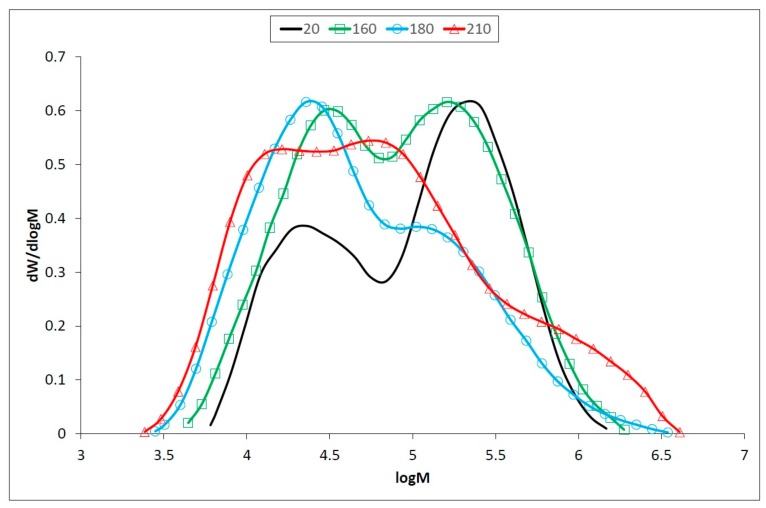
Molecular weight distribution of oak wood holocellulose before and after thermal treatment.

In holocellulose samples, a significant drop in degree of polymerization (DP) occur at 160 °C, by the simultaneous splitting of longer chains of cellulose to shorter hemicelluloses-like chains (Table 2, Figure 5). However, hemicelluloses degraded more rapidly, and the decrease of non-glucose saccharides was observed in thermally treated oak wood [4]. This process is more evident at the temperature of 180 °C leading to a decrease of DP and an increase of polydispersity. In addition to the cleavage of the polysaccharide chains, crosslinking reactions take place at 210 °C, with DP and polydispersity increases (Table 2). A similar phenomenon was observed in the accelerated aging of newsprint paper, which contained about 12% hemicelluloses [17]. Crosslinking in thermally degraded celluloses can take two forms: hydrogen bonding between adjacent chains, or the formation of covalent bridges which join chains together. The effects of crosslinking, particularly covalent crosslinking is to counteract the drop in DP arising from chain scission through network formation [59].

Apart from cellulose, where DP decreases at the temperature of 210 °C, DP in holocellulose increases up to DP approx. 1700 (Table 2, Figure 5).

**Figure 6:**
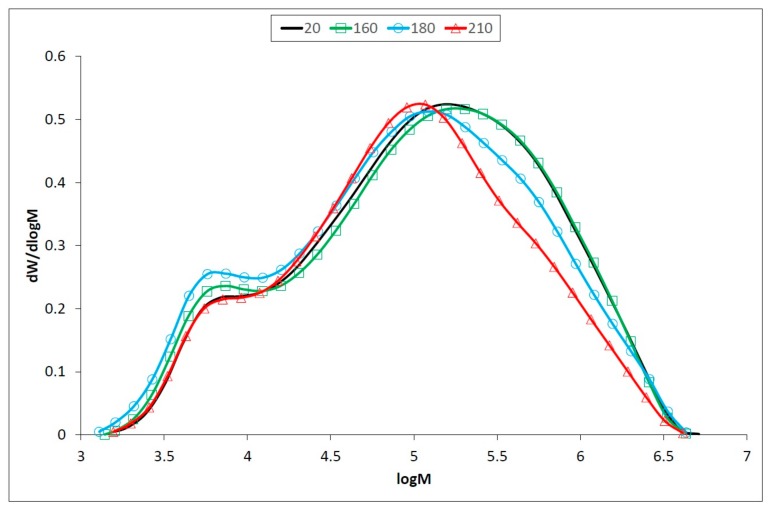
Molecular weight distribution of oak wood cellulose before and after thermal treatment.

Cellulose is relatively stable to thermal treatment. However, minor degradation occurs at relatively low temperatures [60], but it is not as strongly affected as the hemicelluloses. The results of SEC analyses (Table 3, Figure 6) show that the temperature of 160 °C affects the length of the cellulose chain only slightly and the changes in degree of polymerization (DP) are not significant. At 180 °C, the high molecular weight cellulose fractions cleave, and the proportion of low molecular weight fractions increases. This effect continues at 210 °C, whereby low-molecular chains are also cleaved, and DP is 20% lower than for untreated samples [61]. At elevated temperatures, an important amount of acetic acid is released via deacetylation of hemicelluloses which catalyzes depolymerization of the less ordered carbohydrates as hemicelluloses and amorphous cellulose [62,63]. This causes a decrease of DP and an increase of crystallinity.

## 4. Conclusions

The influence of the thermal modification on the chemical changes of oak wood polysaccharides was investigated. Based on Fourier transform infrared spectroscopy and size exclusion chromatography, it can be concluded that thermal treatment leads to an increase of carbonyl groups in lignin together with splitting of aliphatic side chains and demethoxylation. 

The predominance of lignin degradation is evident at lower temperatures, higher temperatures cause mostly condensation reactions and molecular weight increases. Degradation of hemicelluloses resulted in deacetylation and released acetic acid catalyzes hydrolysis of polysaccharide chains. This process is more evident at the temperature of 180 °C leading to a decrease of molecular weight and increase of polydispersity. In addition to the cleavage of the polysaccharide chains, crosslinking reactions take place at 210 °C, with molecular weight and polydispersity increasing.

These structural changes in the main wood components have significant influences on various properties of thermally modified wood. Thermally treated wood is characterized by higher resistance to biotic pests and increases its dimensional and shape stability. According to some sources cited, the thermal modification leads to a reduction in the mechanical strength, which can cause a problem in wood structures damaged by fire. This knowledge should be considered when using thermally treated wood in the design of stressed timber structures.

## Figures and Tables

**Table 1:** Size exclusion chromatography (SEC) results of oak wood lignin before and after thermal treatment ^a,b^.

*T* (°C)	*M*_n_	*M*_w_	*M*_z_	*M*_z+1_	PDI
20	3474 (19)	9216 (138)	23,004 (672)	47,401 (1786)	2.65 (0.03)
160	2987 (34)	8477 (22)	23,838 (97)	54,001 (310)	2.84 (0.03)
180	2736 (23)	7650 (24)	24,899 (644)	63,155 (1626)	2.80 (0.03)
210	3021 (46)	8435 (20)	21,517 (268)	44,751 (1075)	2.79 (0.05)

^a^ Standard deviation values are in parentheses. ^b^ *M*_n_ = number average molecular weight (MW), *M*_w_ = weight-average MW, *M*_z_ = **z** average MW, *M*_z+1_ = z + 1 average MW, PDI (polydispersity index) = *M*_w_/*M*_n._

**Table 2:** SEC results of oak wood holocellulose before and after thermal treatment ^a,b^.

*T* (°C)	*M*_n_	*M*_w_	*M*_z_	*M*_z+1_	PDI	DP
20	51,097 (542)	212,995 (8405)	461,299 (33,735)	699,959 (65,649)	4.17 (0.18)	1315 (52)
160	44,400 (577)	186,382 (3793)	487,413 (13,473)	817,872 (33,397)	4.20 (0.09)	1151 (23)
180	27,524 (353)	146,648 (8298)	640,517 (33,698)	1,339,376 (61,086)	5.33 (0.25)	905 (51)
210	29,042 (327)	275,447 (12,292)	1,223,920 (28,533)	1,929,683 (24,264)	9.48 (0.35)	1700 (76)

^a^ Standard deviation values are in parentheses. ^b^ *M*_n_ = number average molecular weight (MW), *M*_w_ = weight-average MW, *M*_z_ = **z** average MW, *M*_z+1_ = z + 1 average MW, PDI (polydispersity index) = *M*_w_/*M*_n_, DP = degree of polymerization.

**Table 3:** SEC results of oak wood cellulose before and after thermal treatment ^a,b^.

*T* (°C)	*M*_n_	*M*_w_	*M*_z_	*M*_z+1_	PDI	DP
20	39,375 (1165)	385,896 (2376)	1,150,316 (14,034)	1,808,690 (26,230)	9.81 (0.31)	2382 (15)
160	35,634 (438)	384,908 (607)	1,130,145 (4320)	1,758,540 (18,341)	10.80 (0.14)	2376 (4)
180	30,154 (226)	355,894 (1091)	1,190,572 (4984)	1,907,652 (8892)	11.80 (0.10)	2197 (7)
210	33,560 (232)	306,850 (2385)	1,044,946 (10,935)	1,728,619 (19,617)	9.14 (0.13)	1894 (15)

^a^ Standard deviation values are in parentheses. ^b^ *M*_n_ = number average molecular weight (MW), *M*_w_ = weight-average MW, *M*_z_ = **z** average MW, *M*_z+1_ = z + 1 average MW, PDI (polydispersity index) = *M*_w_/*M*_n_, DP = degree of polymerization.
